# Responsiveness of the autonomic nervous system during paced breathing and mental stress in migraine patients

**DOI:** 10.1186/s10194-015-0567-8

**Published:** 2015-09-16

**Authors:** Veronika Rauschel, Andreas Straube, Frank Süß, Ruth Ruscheweyh

**Affiliations:** University of Munich, Department of Neurology, Feodor-Lynen-Straße 19 Marchioninistr 15, 81377 Munich, Germany; University of Dresden, Institute and Policlinic of Occupational and Social Medicine, Fetscherstr, 74 01307 Dresden, Germany

**Keywords:** Migraine, Autonomic nervous system, Heart rate variability, Paced breathing, Mental stress, Skin conductance response, Stress rating, Heart rate, Blood pressure

## Abstract

**Background:**

Migraine is a stress-related disorder, suggesting that there may be sympathetic hyperactivity in migraine patients. However, there are contradictory results concerning general sympathetic activation in migraine patients. To shed more light on the involvement of the autonomic nervous system (ANS) in migraine pathophysiology, we investigated cardiac and cardiovascular reactions during vagal (paced breathing) and sympathetic activation (mental stress test).

**Methods:**

Heart rate variability parameters and skin conductance responses were recorded interictally in 22 episodic migraine patients without aura and 25 matched controls during two different test conditions. The paced breathing test consisted of a five-minute baseline, followed by two minutes of paced breathing (6 breathing cycles per minute) and a five-minute recovery phase. The mental stress test consisted of a five-minute baseline, followed by one minute of stress anticipation, three and a half minutes of mental stress and a five-minute recovery phase. Furthermore we measured blood pressure and heart rate once daily over 2 weeks. Subjects rated their individual current stress level and their stress level during paced breathing and during the mental stress test.

**Results:**

There were no significant differences between migraine patients and controls in any of the heart rate variability parameters in either time domain or frequency domain analysis. However, all parameters showed a non-significant tendency for larger sympathetic activation in migraine patients. Also, no significant differences could be observed in skin conductance responses and average blood pressure. Only heart rates during the 2-week period and stress ratings showed significantly higher values in migraine patients compared to controls.

**Conclusions:**

Generally there were no significant differences between migraine patients and controls concerning the measured autonomic parameters. There was a slight but not significant tendency in the migraine patients to react with less vagal and more sympathetic activation in all these tests, indicating a slightly changed set point of the autonomic system. Heart rate variability and blood pressure in migraine patients should be investigated for longer periods and during more demanding sympathetic activation.

## Background

Migraine attacks consist of a complex sequence of symptoms. In the prodromal phase, vegetative symptoms like hunger, sleepiness and orthostatic hypotension are reported. Later, in the headache phase, vomiting and nausea are typical vegetative symptoms. These symptoms illustrate the strong relationship between migraine and the function of the autonomic nervous system [1]. Furthermore, recently it was reported that transcutaneous stimulation of the parasympathetic nerve system via the vagus nerve can abort migraine attacks [2].

Besides the influence of parasympathetic activity on migraine, there are also several observations which underline the importance of sympathetic activity for migraine triggering. Patients often report that sleep deficits, skipping a meal or stressful situations can trigger their migraine attacks [3, 4]. An increased basal sympathetic tone is also suggested by the fact that migraine is associated with more frequent history of hypertension in epidemiological investigations [5]. Hypertension may be a result of a lasting sympathetic activation [6]. However, other studies have found increased diastolic but not systolic blood pressure in migraine [7] or even an association of migraine with lower blood pressure [6, 8], so the exact relationship between migraine and blood pressure is not clear yet.

Though, research directly trying to quantify abnormalities of the autonomic nervous system in migraine patients delivered quite contradictory results. Besides sympathetic hypofunction [9] or, on the other hand, increased sympathetic activation [10], studies also reported parasympathetic hypofunction [11] or hyperfunction [12].

A possible reason for these striking differences might be that many studies did not define the time point of measurement in relation to the previous or next migraine attack, since migraine seems to involve endogenous rhythms which may also influence the autonomic nervous system [13, 14]. In addition, most studies did not use specific stimulation of autonomic responses. Therefore we decided to measure heart rate variability and skin conductance responses during parasympathetic activation (paced breathing) and sympathetic activation (mental stress) in migraine patients during the interictal period (no migraine for 48 hours before and after the measurement) and controls. In addition we assessed blood pressure and heart rate once daily for 2 weeks.

## Methods

### Subjects

Twenty-five controls and 25 episodic migraine patients without aura were recruited using advertisements on the hospital and university campus and via the internet. Migraine, and the absence of other headache types (e.g. tension type headache), was diagnosed according to the International Classification of Headache Disorders (ICHD-III) [15] by an experienced physician from the outpatient headache clinic at the Department of Neurology. Healthy participants had to be free of any headaches. In addition, migraine participants had to be free of any preventive medication for at least 4 weeks prior to the experiment. Migraine patients were tested interictally, meaning they were free of headache for at least 48 hours before and after the experimental session which was confirmed by a diary and a telephone call 48 h after the recordings. Analgesics and triptans were not allowed within 48 hours before and after the experiment. Participants were asked not to consume alcohol or caffeinated drinks for 3 hours prior to the experiment. Since three migraine patients developed headache within 48 hours after the experimental session and therefore had to be excluded from analysis, only 22 migraine patients (age 28.1 ± 6.9; male/female 1:21) and 25 controls (age 26.1 ± 7.1; male/female 3:22) could be analyzed. Demographic data is given in Table 1. The study was approved by the local ethics committee and conducted in accordance with the Declaration of Helsinki. Subjects provided written informed consent before participation.

**Table 1:** Characteristics of the subjects

	Migraine	Control
n	22	25
Age	28.1 ± 6.9	26.1 ± 7.1
Gender (male:female)	1:21	3:22
Headache history (years)	10.7 ± 6.1	
Headache days/month	4.9 ± 2.2	
Headache intensity (1-10)	6.8 ± 1.3	

All migraine patients suffered from episodic migraine without aura. Values are mean ± SD

### Experimental design and data acquisition

The electrocardiogram (ECG), skin conductance and respiratory excursions were measured using the SUEmpathy100 system (SUESS Medizin-Technik GmbH, Mittelstraße 9, 08280 Aue, Germany). The ECG was recorded with a sampling rate of 512 Hz using three electrodes (Ag/AgCl), attached over the medioclavicular line over the first intercostal space (left and right) and over the anterior axillar line over the fifth left intercostal space. Respiratory excursions were obtained by a sensor attached to the inferior aperture of the thorax (only during the paced breathing test, see below). Skin conductance response was measured using Ag/AgCl electrodes attached to the second and third finger of the left hand (only during the mental stress test, see below).

Data were obtained with patients in a relaxed supine position in a silent and darkened room. The room was kept at a constant temperature of 22 °C ± 2 °C. Two experiments were always conducted at the same time in the early evening. The first experiment (paced breathing test) consisted of a five-minute baseline, followed by two minutes of paced breathing (6 breathing cycles per minute, paced by standardized, pre-recorded instructions administered via earphones) and a five-minute recovery phase. The second experiment (mental stress test) consisted of a five-minute baseline, followed by one minute of anticipation, three and a half minutes of mental stress and a five-minute recovery phase. During the anticipation phase, subjects were told that the stress test would be an arithmetic test requiring them to repeatedly subtract the same two-digit number from a four-digit number (e.g. 1750–13) as fast as possible. During the mental stress phase, subjects performed the task under the surveillance of the investigator.

In the 2 weeks preceding or following the experimental session, subjects measured their blood pressure and heart rate daily (in the morning, after waking up) using an Omron M500 blood pressure monitor (Omron Healthcare Europe B.V., Scorpius 33, 2132 LR Hoofddorp, P.O.Box 2050 2130 GL Hoofddorp, Netherlands). A headache diary was kept during this time.

Data on headache history, number of headache days/month, headache intensity and average stress levels during the preceding 24 hours (quantified on a numerical rating scale from 0–10, where 0 = no stress at all and 10 = most intense stress imaginable) were obtained by a clinical interview. Stress levels were also obtained after each experiment, with respect to the stress levels reached during the mental stress test and during the paced breathing test. We opted for a single self-rating item to assess stress levels because single-item measures are timesaving, easily understandable and have recently demonstrated good reliability and validity [16]. Stress levels were measured on the numerical rating scale (NRS) which is conceptionally similar to the visual analogue scale (VAS) that has recently been validated to assess perceived stress in occupational medicine [17].

### Data analysis

The SUEmpathy100 system automatically analyses the ECG recording during the different experimental phases, providing data on the average heart rate (HR), the coefficient of variation (CV) of the interval between two subsequent R waves (RR interval) as a measure of overall heart rate variability, the root mean square of successive differences in RR intervals (RMSSD) as a measure of short-term heart rate variability, the power spectrum of the heart rate variability in three different frequency bands (very low frequency [VLF]: 0.0033–0.04 Hz, corresponding to predominantly sympathetically mediated activity; low frequency [LF]: 0.04–0.15 Hz, reflecting a mixture of both sympathetic and parasympathetic activity; and high frequency [HF]: 0.15–0.4Hz, corresponding to predominantly parasympathetically mediated activity), and the LF/HF ratio as a measure of sympathetic in relation to parasympathetic activity [18]. After each experiment, ECG traces were manually revised to ensure that the R-waves had been correctly detected by the in-built algorithm. Where the algorithm had failed, R wave detection was corrected manually. Then, data on the parameters listed above and on the skin conductance response (sampling rate: 512 Hz) were exported to Excel for further analysis. To determine the expiration/inspiration ratio of RR intervals during the paced breathing phase, maximum RR intervals during expiration and minimum RR intervals during inspiration were detected during each breathing cycle, averaged over all breathing cycles obtained and the expiration/inspiration ratio was calculated. To analyze the influence of mental stress on skin conductance responses, the average skin conductance response during each experimental phase was calculated and the average skin conductance level during the last minute of the baseline phase was subtracted from this value.

### Statistical analysis

Data were analyzed using the Statistical Package for Social Sciences (SPSS, version 22, IBM Corporation, Armonk, New York, USA). Values are given as mean ± standard deviation (SD) unless otherwise stated. *P* < 0.05 was considered significant. Age and sex were compared between groups using a *t*-test and a chi-square test respectively. The experimental parameters (heart rate, different parameters of the time and frequency domain heart rate analysis, skin conductance responses) were compared between groups using repeated measures ANOVA (within-subject factor: different phases of the two tests [for paced breathing: baseline, paced breathing, and recovery; and for mental stress: baseline, anticipation, stress, and recovery], between-subject factor: group) and t-tests as appropriate. Stress ratings were compared between groups using repeated measures ANOVA (within subject factor: reference [average, paced breathing, mental stress], between subjects factor: group). Average blood pressure and heart rates obtained over 2 weeks were compared between groups using unpaired t-tests. Blood pressure and heart rates between headache days and interictal days in migraine patients were compared using paired t-tests.

## Results

### Subjects

Age and sex were balanced between groups (see Table 1) (age: T [45] = -1.0, *p* = 0.32, sex: *χ*2[1] = 0.8, *p* = 0.36).

#### Blood pressure and heart rate (2 week average)

Average blood pressure and heart rate, derived from daily measurements over 2 weeks are shown in Table 2 together with corresponding statistics. Unpaired t-tests showed no significant differences in systolic or diastolic blood pressure (*p* = 0.43). However, average heart rate was slightly but significantly elevated in migraine patients as compared to controls (75.5 bpm in migraine patients and 70.9 bpm in controls, *p* = 0.03), in agreement with the idea of higher sympathetic and/or lower parasympathetic activity in migraine patients as compared to controls. During the two weeks of blood pressure and heart rate measurement, migraine patients suffered from headache for on average 2.55 ± 1.77 days. We tested if blood pressure and/or heart rate were different on headache days, compared to interictal days, but there were no significant differences. (BP systolic : mean ± SD: ictal = 111.4 ± 9.2, interictal = 111.6 ± 8.9; T [19] = -0.19, *p* = 0.85; BP diastolic: mean ± SD: ictal = 73.4 ± 12.3, interictal = 72.8 ± 8.0; T [19] = 0.42, *p* = 0.68; HR : mean ± SD: ictal = 76.5 ± 9.8, interictal = 75.1 ± 8.1; T [19] = 0.96, *p* = 0.35).

**Table 2:** Average blood pressure and heart rate over 2 weeks

Parameter		Migraine (n = 22) Mean ± SD	Control (n =25) Mean ± SD	Group comparison
BP [mmHg]	Systolic blood pressure	112.53 ± 10.68	116.79 ± 10.15	T [45] = 1.4 *p* = 0.17
	Diastolic blood pressure	72.76 ± 6.98	71.14 ± 6.78	T [45] = -0.8 *p* = 0.43
HR [bpm]		75.51 ± 7.42	70.85 ± 6.64	T [45] = -2.3 *p* = 0.03

Values are mean ± SD. *BP* blood pressure, *HR* heart rate, Results of unpaired t-tests are given

#### Stress ratings

**Fig. 1:**
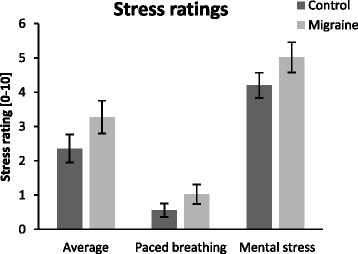
Stress self-ratings average stress level, during the paced breathing and during the mental stress test. Ratings for interictal migraine patients (*n* = 22) and controls (*n* = 25) are illustrated as mean ± SEM. The y-axis illustrates the stress rating scale (0-10)

Subjective stress ratings were obtained with reference to average stress levels (during the 24 hours preceding measurement), and stress levels during the mental stress and the paced breathing conditions (see Table 3 and Fig. 1). There was a main effect of group (*p* = 0.029), indicating elevated subjective stress ratings in migraine patients compared to controls in all conditions, but no significant interaction between condition and group (*p* = 0.47, see Table 3 for statistics).

**Table 3:** Comparison of stress ratings between controls and migraine patients

Parameter		Migraine (*n* = 22) Mean ± SD	Control (*n* =25) Mean ± SD	Main effect of block	Main effect of group	Interaction
Stress ratings [0-10]	Average during preceding 24 h	3.27 ± 2.25	2.36 ± 2.04	F [2,44] = 103.7 *p* < 0.001	F [1] = 5.1 *p* = 0.029	F [2,44] = 0.8 *p* = 0.47
	During paced breathing	1.09 ± 1.51	0.56 ± 0.96			
	During mental stress	5.41 ± 1.97	4.20 ± 1.85			

Values are mean ± SD. Results of ANOVA are given

#### Heart rate analysis during the paced breathing test

**Fig. 2:**
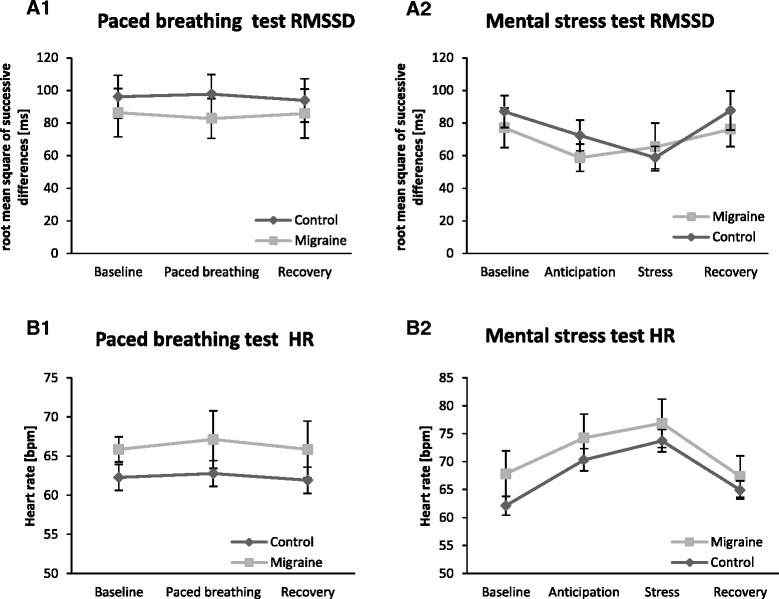
Group differences of cardiovascular parameters during paced breathing and mental stress test between controls and migraine patients. A RMSSD (*root mean square of successive differences*) and B HR (*heart rate*) profiles are illustrated interictally for migraine patients (*n* = 22) and controls (*n* = 25) as mean ± SEM. **a1** RMSSD during the paced breathing test. **a2** RMSSD during the mental stress test. **b1**Heart rate (*HR*) during the paced breathing test. **b2** HR during the mental stress test

Results of heart rate and heart rate variability parameters during the paced breathing test in controls and migraine patients are summarized in Table 4 together with the corresponding statistics. RMSSD (root mean square of successive differences) and heart rate profiles during the paced breathing test are illustrated in Fig. 2 (a1 and b1). Average heart rate (*p* = 0.014) and all heart rate variability parameters (VC: *p* < 0.001, VLF power: *p* < 0.001, LF power: *p* < 0.001, HF power: *p* = 0.003, LF/HF: *p* < 0.001), with the exception of the RMSSD (*p* = 0.89), showed a main effect of block, indicating that paced breathing had a significant effect on these parameters. There was no significant main effect of group (VC: *p* = 0.60, RMSSD: *p* = 0.56, VLF power: *p* = 0.85, LF power: *p* = 0.98, HF power: *p* = 0.78, LF/HF: *p* = 0.92) and no significant interaction between block and group for any of the parameters. However, when looking at the raw values in Table 4, it becomes clear that migraine patients show a slightly (statistically non-significant) higher sympathetic and/or lower parasympathetic tone for all parameters investigated (smaller VC and RMSSD, increased VLF power, reduced HF power, increased heart rate). A trend of significance (*p* = 0.09) was found for heart rate. Consistent with the finding in the 2-week heart rate averages described above, heart rate was again higher in migraine patients. E/I ratio (ratio of the heart rates during inspiration and expiration) was assessed only during the paced breathing block and did not differ between the groups (*p* = 0.82).

**Table 4:** Comparison of heart rate and heart rate variability parameters during the paced breathing test between controls and migraine patients

Parameter	Block	Migraine (n = 22) Mean ± SD	Control (n =25) Mean ± SD	Main effect of block	Main effect of group	Interaction block*group
VC [%]	Baseline	8.69 ± 4.85	9.10 ± 3.83	**F [2,44] = 27.7** ***p*** ** < 0.001**	F [1] = 0.3 *p* = 0.60	F [2,44] = 0.2 *p* = 0.83
	Paced breathing	11.46 ± 5.10	12.37 ± 3.84			
	Recovery	8.83 ± 4.73	9.42 ± 3.55			
RMSSD [ms]	Baseline	86.27 ± 69.69	96.07 ± 65.66	F [2,44] = 0.113 *p* = 0.89	F [1] = 0.4 *p* = 0.56	F [2,44] = 0.4 *p* = 0.69
	Paced breathing	82.75 ± 57.25	97.71 ± 60.28			
	Recovery	85.84 ± 70.83	93.86 ± 66.38			
VLF power [ms^2^]	Baseline	77 ± 111	72 ± 83	**F [2,44] = 25.6** ***p*** ** < 0.001**	F [1] = 0.04 *p* = 0.85	F [2,44] = 0.06 *p* = 0.94
	Paced breathing	215 ± 235	200 ± 134			
	Recovery	78 ± 109	78 ± 77			
LF power [ms^2^]	Baseline	3450 ± 4011	3467 ± 3738	**F [2,44] = 25.7** ***p*** ** < 0.001**	F [1] = 0.001 *p* = 0.98	F [2,44] = 0.06 *p* = 0.94
	Paced breathing	10354 ± 9916	10600 ± 6637			
	Recovery	4369 ± 5454	4215 ± 3937			
HF power [ms^2^]	Baseline	4252 ± 7822	4457 ± 6130	**F [2,44] = 6.6** ***p*** ** = 0.003**	F [1] = 0.08 *p* = 0.78	F [2,44] = 0.3 *p* = 0.77
	Paced breathing	2043 ± 3378	2296 ± 3335			
	Recovery	3323 ± 5754	4166 ± 6971			
LF/HF	Baseline	1.46 ± 1.35	1.43 ± 1.19	**F [2,44] = 25.1** ***p*** ** < 0.001**	F [1] = 0.01 *p* = 0.92	F [2,44] = 1.3 *p* = 0.29
	Paced breathing	13.58 ± 14.11	13.46 ± 11.49			
	Recovery	1.81 ± 1.57	2.36 ± 2.40			
E/I	Paced breathing	1.37 ± 0.20	1.38 ± 0.17	-	T [44] = 0.2 p = 0.82	-
HR [bpm]	Baseline	65.86 ± 7.45	62.28 ± 8.34	**F [2,44] = 4.7** ***p*** ** = 0.014**	F [1] = 3.1 *p* = 0.09	F [2,44] = 0.6 *p* = 0.58
	Paced breathing	67.12 ± 7.00	62.77 ± 8.27			
	Recovery	65.84 ± 7.15	61.91 ± 8.39			

Values are mean ± SD. *VC* Coefficient of variation in percent (SD/RR*100), RMSSD: root mean square of successive differences = root mean square of the sum of squares of differences between adjacent RR (normalized R-to-R) intervals, *VLF* power in the very low frequency band (0.0033–0.04Hz), *LF* power in the low frequency band (0.04–0.15Hz), *HF* power in the high frequency band (0.15–0.4Hz), LF/HF: ratio of LF to HF, *E/I*: heart rate ratio between expiration and inspiration, *HR* heart rate. Results of ANOVA and unpaired t-tests are given. Significant effects are marked in bold. There were no significant group differences

#### Heart rate analysis during the mental stress conditions

Heart rates and heart rate variability during the mental stress test were measured and are reported in Table 5 together with corresponding statistics. RMSSD and heart rate profiles during the mental stress test are illustrated in Fig. 2 (a2 and b2). Again, heart rate and all heart rate variability parameters showed a main effect of block (see Table 5, VC: *p* = 0.015, RMSSD: *p* = 0.019, VLF power: *p* = 0.017, LF power: *p* = 0.023, HF power: *p* = 0.006, LF/HF: *p* = 0.033, HR: *p* < 0.001), indicating that the mental stress paradigm did indeed significantly affect these parameters. But no significant main effect of group (VC: p = 0.49, RMSSD: p = 0.57, VLF power: *p* = 0.61, LF power: *p* = 0.84, HF power: *p* = 0.21, LF/HF: *p* = 0.65, HR: *p* = 0.15) and no significant interaction between block and group were found for all parameters during the mental stress test. Again, the majority of parameters (VC, RMSSD, HF power, and HR) showed a slightly (but non-significant) stronger sympathetic activation and/or parasympathetic hypofunction in migraine patients compared to controls.

**Table 5:** Comparison of heart rate, heart rate variability and skin conductance level parameters during the mental stress test between controls and migraine patients

Parameter	Phase	Migraine (n = 22) Mean ± SD	Control (n =25) Mean ± SD	Main effect of block	Main effect of group	Interaction
VC [%]	Baseline	8.35 ± 3.99	8.80 ± 3.59	**F [3,43] = 3.9** ***p*** ** = 0.015**	F [1] = 0.5 *p* = 0.49	F [3,43] = 1.1 *p* = 0.37
	Anticipation	8.53 ± 3.41	10.20 ± 3.84			
	Stress	9.41 ± 6.13	9.44 ± 3.98			
	Recovery	9.50 ± 4.16	10.36 ± 5.78			
RMSSD [ms]	Baseline	77.03 ± 56.39	87.01 ± 49.41	**F [3,43] = 3.7** ***p*** ** = 0.019**	F [1] = 0.3 *p* = 0.57	F [3,43] = 0.8 *p* = 0.52
	Anticipation	58.77 ± 39.45	72.35 ± 47.23			
	Stress	65.31 ± 68.44	58.76 ± 34.66			
	Recovery	76.16 ± 49.86	87.65 ± 59.97			
VLF power [ms^2^]	Baseline	58 ± 85	62 ± 69	**F [3,43] = 3.8** ***p*** ** = 0.017**	F [1] = 0.3 *p* = 0.61	F [3,43] = 1.1 *p* = 0.35
	Anticipation	24 ± 27	44 ± 44			
	Stress	33 ± 34	35 ± 39			
	Recovery	65 ± 88	68 ± 63			
LF power [ms^2^]	Baseline	3496 ± 4722	3126 ± 3192	**F [3,43] = 3.5** ***p*** ** = 0.023**	F [1] = 0.04 *p* = 0.84	F [3,43] = 1.5 *p* = 0.22
	Anticipation	1802 ± 1978	2889 ± 2512			
	Stress	2239 ± 2172	2293 ± 2001			
	Recovery	3865 ± 4813	3720 ± 3062			
HF power [ms^2^]	Baseline	2241 ± 2611	3645 ± 4606	**F [3,43] = 4.8** ***p*** ** = 0.006**	F [1] = 1.6 *p* = 0.21	F [3,43] = 1.6 *p* = 0.21
	Anticipation	1021 ± 1049	1595 ± 1663			
	Stress	981 ± 976	1632 ± 2213			
	Recovery	2397 ± 2774	3133 ± 4121			
LF/HF [Hz]	Baseline	1.74 ± 1.18	1.73 ± 1.93	**F [3,43] = 3.2** ***p*** ** = 0.033**	F [1] = 0.2 p = 0.65	F [3,43] = 1.2 p = 0.34
	Anticipation	2.55 ± 1.80	2.64 ± 1.77			
	Stress	2.91 ± 1.97	2.10 ± 1.19			
	Recovery	2.04 ± 1.41	2.12 ± 1.77			
HR [bpm]	Baseline	67.83 ± 11.51	62.10 ± 8.48	**F [3,43] = 58.9** ***p*** ** < 0.001**	F [1] = 2.2 *p* = 0.15	F [3,43] = 1.3 *p* = 0.30
	Anticipation	74.23 ± 10.30	70.31 ± 9.94			
	Stress	76.86 ± 9.83	73.72 ± 10.00			
	Recovery	67.36 ± 6.88	64.93 ± 8.05			
SCR [μS]	Anticipation	2.72 ± 2.53	2.38 ± 2.50	**F [3,43] = 32.5** ***p*** ** < 0.001**	F [1] = 0.2 *p* = 0.70	F [3,43] = 0.2 *p* = 0.91
	Stress	5.43 ± 3.72	4.98 ± 3.28			
	Recovery	2.40 ± 1.83	2.38 ± 1.87			

Values are mean ± SD. *VC* Coefficient of variation in percent (SD/RR*100), RMSSD: root mean square of successive differences = root mean square of the sum of squares of differences between adjacent RR (normalized R-to-R) intervals, VLF power in the very low frequency band (0.0033–0.04Hz), LF: power in the low frequency band (0.04-0.15Hz), HF: power in the high frequency band (0.15–0.4Hz), LF/HF ratio of LF to HF, HR: heart rate, SCR: average skin conductance response in each phase. Results of ANOVA and unpaired t-tests are given. Significant effects are marked in bold. There were no significant group differences

### Skin conductance

**Fig. 3:**
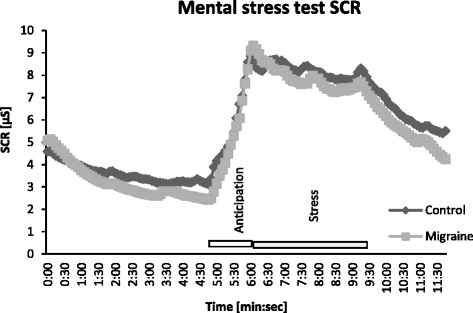
Group differences in skin conductance latency (*SCL*) during the mental stress test. SCL profiles for interictal migraine patients (*n* = 22) and controls (*n* = 25) are illustrated as mean ± SEM. During the anticipation (*minute 5-6*) subjects were instructed what the stress test will be, followed by 2.5 minutes stress test

The skin conductance responses were only assessed during the mental arithmetic test (Table 5 and Fig. 3). There was a main effect of block (*p* < 0.001), but no main effect of group (*p* = 0.70), and no interaction between group and block (*p* = 0.91).

## Discussion

The main result of our study assessing cardiac and cardiovascular parameters during experimental activation of the parasympathetic and sympathetic systems and during daily life over a period of 2 weeks is that there were no striking differences in these parameters between episodic migraine patients without aura and matched controls. However, there may be subtle differences as revealed by a significantly elevated average heart rate over 2 weeks, higher subjective stress ratings, and a trend towards increased heart rate during the paced breathing condition in migraine patients. It is worth discussing if these subtle findings indicate a slight shift to more sympathetic nervous system activity in migraine patients compared to controls.

There are a number of earlier studies dealing with autonomic nervous system activity in migraine. Overall, they do not present a clear picture of the possible alterations of the autonomic nervous system in migraine. There are a number of studies which postulate a sympathetic hyperfunction and/or a sympathetic hypofunction in migraine. Similar to our results, Gass and Glaros (2013) found a sympathetic hyperfunction in migraine patients [10]. They analyzed heart rate variability, skin temperature, skin conductance, and respiration in 21 patients with tension-type and/or migraine headache and 19 controls during a 5-min resting period. There were no statistical differences in HRV measures in the frequency domain, or in skin temperature or skin conductance between the groups, but the number of consecutive R-to-R intervals that varied by more than 50 ms were significantly lower in the headache group than in the control group. They concluded that these results suggest increased sympathetic and/or decreased parasympathetic nervous system activity in headache. Yerdelen et al. examined heart rate recovery after physical exercise as an index for vagal activity in migraine and tension-type headache patients (TTH) and controls [19]. Heart rate recovery was similar in all three groups, but resting heart rates in migraine were higher than in episodic TTH, albeit not significantly different from controls. This result is somewhat similar to our finding during the consecutive measurement for 2 weeks. They conclude that sympathetic tone in migraine patients is elevated compared to patients with episodic tension-type headache.

Similarly to our study, where there was a stronger group difference in subjective measures (stress levels) than objective measures, Tome-Pires et al. found only a trend for higher skin conductance responses for pain descriptors and emotional words in migraine patients compared to controls, but a higher recall of emotional words in the migraine group [20].

Another study examined heart rate variability for a longer period. They recorded a forty-eight-hour Holter electrocardiogram in 27 migraine patients in the headache-free period and 24 healthy controls during normal daily activity. They found significant differences in circadian rhythm in different heart rate variability parameters between migraine patients and controls, pointing towards parasympathetic hypofunction in migraine patients [11]. The study by Thomson and colleagues in which cardiovascular reflexes in response to the Valsalva manoeuvre were measured in migraine patients also pointed towards a parasympathetic hypofunction [21].

Some studies also failed to find differences between migraine patients and controls, or signs of sympathetic hypofunction in migraine. For instance, no differences in heart rate, temporal artery pulse volume and skin conductance response between migraine patients and controls during self-selected ‘stressful’ and ‘relaxing’ imagery were found [22]. Cambron et al. report evidence for a subtle pupillary sympathetic hypofunction in migraine patients, observed as a prolonged latency to light reflex, which is revealed after the administration of apraclonidine [9].Similarly, resting supine sympathetic hypofunction as measured by heart rate variability parameters in migraine patients with aura was reported [23].

In spite of the heterogeneity of the published results, the most often discussed interpretations concerning the alteration of autonomic functions in migraine patients in the literature are:

1. a sympathetic hyperactivation and/or
2. a parasympathetic hypoactivation.

The present result that heart rates over 2 weeks are increased in migraine patients may support the hypothesis of increased sympathetic and/or impaired parasympathetic activity in migraine. Consistently, there was also a trend for higher heart rates in migraine patients during all phases of the paced breathing test (*p* = 0.09) and the mental stress test (0.15). Heart rate is regulated by both the sympathetic and the parasympathetic nervous system, so an increased heart rate in migraine patients could also be interpreted as an increased sympathetic tone or, alternatively, reduced parasympathetic activity. Moreover, when looking at the raw values, there was a slight shift towards less sympathetic and/or more parasympathetic activity for almost every parameter assessed (Tables 4 and 5), although this did not reach significance. For example, the RMSSD and the high-frequency band power, which are measures of parasympathetic activity, were consistently lower in migraine patients than in healthy controls during all phases of the paced breathing and mental stress tasks.

Furthermore, it was shown that migraine patients feel more stressed in all conditions tested (in general, during paced breathing and the mental stress test) .This confirms earlier findings that migraine patients tend to react towards environmental factors more and with a lower stimulus threshold [24, 25], possibly associated with a higher sympathetic tone. Such increased basic sympathetic activity may also account for the fact that in epidemiological population-based studies migraine patients tend to have a higher average blood pressure [5].

### Strengths and limitations of our study

Certainly, the strength of our study is the careful selection and monitoring of the migraine patients, ensuring that all patients were recorded interictally and were free of preventive drugs or recent intake of analgesics. The migraine patients included in the present study had, on average, about 5 headache days per month (range: ±2.2). We cannot rule out the possibility that migraine patients suffering from headache more frequently exhibit a stronger alteration of the autonomic nervous system, but then it becomes almost impossible to obtain interictal recordings. As we only obtained a single interictal recording, it can also not be ruled out that autonomic function parameters and their reactivity to the paced breathing and mental stress tests change during the migraine cycle, as it was shown for the contingent negative variation (CNV)[26]. The only significant difference between patients and controls in the present study was a slightly increased heart rate in patients over the two weeks interval. We showed that this was not due to increased heart rate during migraine attacks. However, because of frequent incapacitating headaches, migraine patients may also differ from controls in other factors not assessed in the present study, such as body mass index or physical training level. These factors might explain differences in heart rate, and should be considered in future studies.

## Conclusion

The involvement of the autonomic nervous system in migraine pathophysiology is complex and heterogeneous. Numerous (but not all) published results suggest a sympathetic hyperactivation or parasympathetic hypoactivation. Our results point in the same direction with a slight difference in the balance between sympathetic and parasympathetic activation with a net increased sympathetic tone. Further long-term studies in a larger group of migraine patients will be needed to investigate heart rate variability parameters in migraine patients during daily routine and during all phases of the migraine cycle.

## Ethics or institutional review board approval

The study was approved by the local ethics committee at the University of Munich (Project number: 133-13).Participants gave informed consent before taking part.

## Abbreviations

ANS: Autonomic nervous system
CNS: Central nervous system
ICHD: International classification of headache disorders
ECG: Electrocardiogram
HR: Heart rate
VC: Coefficient of variation
RMSSD: Root mean square of successive differences
VLF: Power in the very low-frequency band
LFN: Low-frequency noise
HFN: High-frequency noise; LF/HF ratio of LFN to HFN
E/I: Heart rate ratio between expiration and inspiration
SCL: Skin conductance latency
SPSS: Statistical package for social sciences
SD: Standard deviation
ANOVA: Analysis of variance
